# Synthesis, Characterization, and Biological Evaluation of Some New Functionalized Terphenyl Derivatives

**DOI:** 10.1155/2012/530392

**Published:** 2012-12-13

**Authors:** Seranthimata Samshuddin, Badiadka Narayana, Balladka Kunhanna Sarojini, Divya N. Shetty, Nalilu Suchetha Kumari

**Affiliations:** ^1^Department of Studies in Chemistry, Mangalore University, Mangalagangotri, Karnataka, Mangalore 574 199, India; ^2^Research Department of Chemistry, P.A. College of Engineering, Nadupadavu, Karnataka, Mangalore 574153, India; ^3^Department of Biochemistry, K. S. Hegde Medical Academy, Deralakatte 574162, India

## Abstract

New functionalized terphenyl derivatives incorporating various heterocyclic rings are prepared by using 4,4′′-difluoro-5′-hydroxy-1,1′:3′,1′′-terphenyl-4′-carbohydrazide as a key intermediate derived from 4,4′-difluoro chalcone, a versatile synthone. All the derivatives are characterized by ^1^H NMR, IR, and mass spectral data. All the synthesized products are screened for their *in vitro* antimicrobial and antioxidant properties. The majority of the tested compounds exhibited significant antioxidant activity and some of them showed good antimicrobial activity.

## 1. Introduction

Terphenyl is a common structural motif found in various natural products, largely isolated from microbes and mushrooms [1]. In recent years, it has been reported that some terphenyls exhibit considerable biological activities, for example, potent anticoagulants, immunosuppressants, antithrombotic, neuroprotective, specific 5-lipoxygenase inhibitory, and cytotoxic activities [2]. Because of their promising biological activities and important properties, terphenyls have been produced increasing research interest. Therefore, synthesis of terphenyl derivatives has been a fascinating area in organic field [3].

Recently we have reported a new and simple method for the preparation of ester derivative of terphenyl starting from 4,4′-difluoro chalcone [4]. The esters can be easily converted into the corresponding carbohydrazide by the reaction with hydrazine hydrate. Hydrazides and related compounds have been described as useful precursors for the assembly of various heterocyclic rings. A large number of hydrazides are reported to be of biological interest [5, 6], while oxadiazole derivatives and thiosemicarbazides have been reported to possess antibacterial [7, 8], antifungal [9, 10], and other biological activities. Furthermore, a number of substituted thiazolines and thiazolidinones are found to exhibit appreciable antimicrobial activities [11–15]. 

In the course of investigation, we have found that 4,4′′-difluoro-5′-hydroxy-1,1′:3′,1′′-terphenyl-4′-carbohydrazide is an excellent building block for the synthesis of numerous heterocyclic systems. In view of the pharmacological importance of terphenyls and in continuation of our work on synthesis of various derivatives of 4,4′-difluoro chalcone [16–23], it was decided to prepare new functionalized terphenyl derivatives by using 4,4′′-difluoro-5′-hydroxy-1,1′:3′,1′′-terphenyl-4′-carbohydrazide as the key intermediate and to study their biological activities.

## 2. Chemistry

**Scheme 1:**
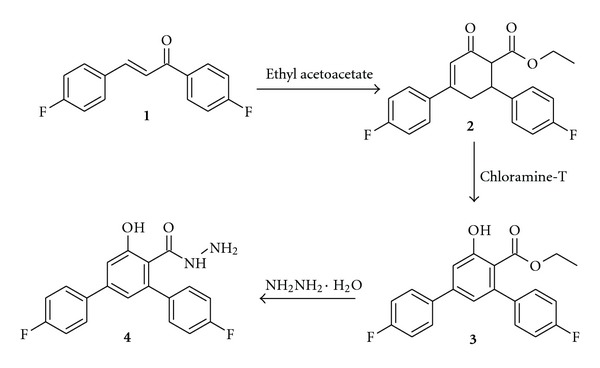


A novel terphenyl derivative, ethyl 4,4′′-difluoro-5′-hydroxy-1,1′:3′,1′′-terphenyl-4′-carboxylate **3**, was synthesized by the oxidative aromatization of cyclohexenone derivative of 4,4′-difluoro chalcone using chloramine-T [4]. The ester **3** was converted into the corresponding carbohydrazide **4** in high yield by the reaction with hydrazine hydrate (Scheme 1). In the IR spectrum of carbohydrazide **4** broad stretching band at 3290 cm^−1^ was due to NH while strong stretching band at 1672 cm^−1^ was attributed to carbonyl. ^1^H NMR spectrum showed two singlets at *δ* 9.19 ppm and *δ* 4.18 ppm which were accounted for NH_2_ and NH protons. The mass spectrum of **4** showed a molecular ion peak at *m*/*z* 341.0 which confirmed its molecular weight.

**Scheme 2:**
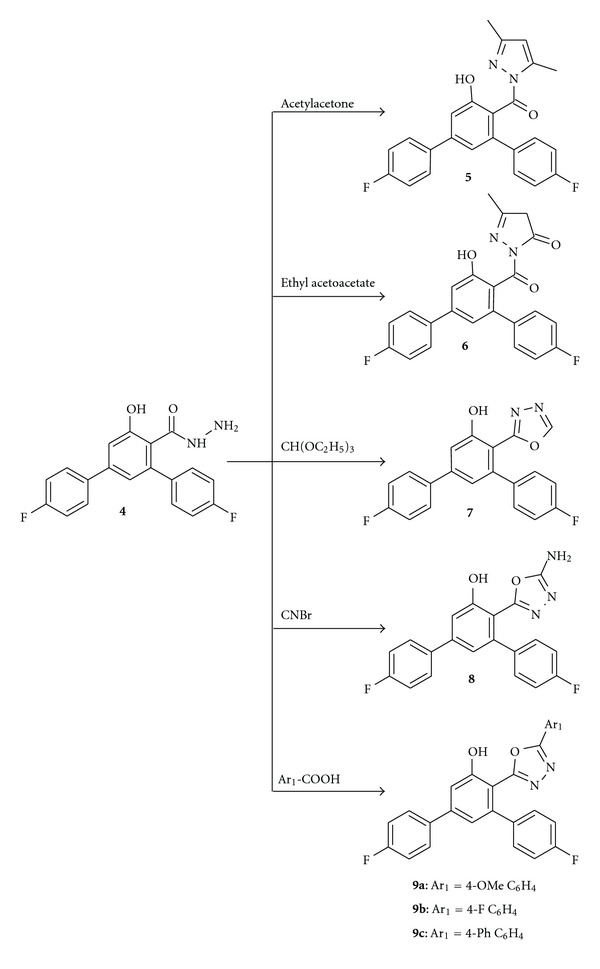


The carbohydrazide is a versatile key intermediate for the synthesis of several heterocyclic systems such as pyrazoles, thiazoles, 1,3,4-oxadiazoles, and 1,2,4-triazoles [24, 25]. Hence, the carbohydrazide **4** was treated with various reagents to afford pyrazole and 1,3,4-oxadiazole derivatives (Scheme 2). Condensation reaction of carbohydrazide **4** with dicarbonyl compounds, namely, acetylacetone and ethyl acetoacetate, in the presence of catalytic amount of triethylamine afforded the corresponding substituted pyrazole derivatives **5** and **6**, respectively. The IR spectrum of pyrazole **5** showed the presence of vibrations at 2926 cm^−1^ due to methyl group. The ^1^H NMR spectrum of **5** showed two singlets at *δ* 2.29 and 2.70 ppm due to three each protons of two methyl groups at C-3 and C-5 carbon of pyrazole ring, respectively, and hence confirming the presence of 3,5-dimethyl-1*H*-pyrazole moiety. Similarly, in the IR spectrum of pyrazole **6**, vibrations at 1654 cm^−1^ indicates the presence of carbonyl group of the pyrazolone nuclei. Singlets at *δ* 2.84 and 2.03 ppm in the ^1^H NMR spectrum were observed for the methyl and methylene protons of the pyrazolone moiety.

Cyclization to the oxadiazoles was carried out by treating the carbohydrazide **4** with triethyl orthoformate/toluene, cyanogen bromide/ethanol and with aromatic acids/phosphorous oxychloride. The IR spectra of oxadiazoles **7** and **9** showed the absence of vibrations due to NH and NH_2_ groups. Their ^1^H NMR spectra also indicated the absence of NH protons, thus confirmed the cyclization reaction. In the IR spectrum of oxadiazole **8**, a stretching vibration observed at 3385 cm^−1^ which indicates the presence of amino group. A singlet at *δ* 6.93 ppm in the ^1^H NMR spectrum of **8** confirmed the presence of NH_2_ group and hence proved that ring closure starting from carbohydrazide **4** resulted in the formation of 5-amino-1,3,4-oxadiazole moiety.

**Scheme 3:**
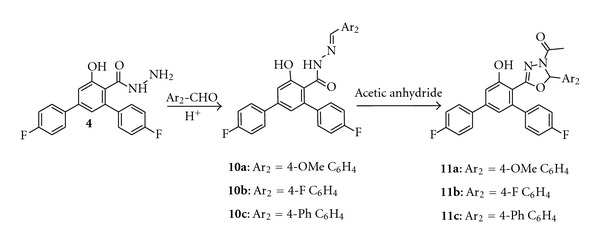


An arylidine hydrazone incorporated into the parent terphenyl was also synthesized. Thus, condensation of carbohydrazide **4** with aromatic aldehydes in absolute ethanol in presence of few drops of concentrated hydrochloric acid afforded the corresponding Schiff bases **10a–c**. The Schiff bases **10a–c** thus obtained were cyclized into 1,3,4-oxadiazole derivatives **11a–c** by refluxing them with acetic anhydride (Scheme 3). IR spectra of **10a–c**, showed an NH stretching band at around 3329 cm^−1^ and a carbonyl absorption band near 1647 cm^−1^. The ^1^H NMR spectrum of **10c**, as an example, showed a singlet signal at *δ* 8.15 ppm corresponding to CH of the benzylidene group and another singlet signal at *δ* 11.71 ppm due to NH proton, hence confirming the formation of Schiff base hydrazone. The IR spectra of compounds **11a–c** had different characteristics since they showed no N–H stretching bands, but carbonyl stretching near 1770 cm^−1^ which could be attributed to the carbonyl of acetyl group. ^1^H NMR spectra of **11a–c** showed a singlet in the spectra in the range *δ* 6.41–6.52 ppm indicating CH resonance of the oxadiazoline ring which is in accordance with the reported values found in the literature [26].

**Scheme 4:**
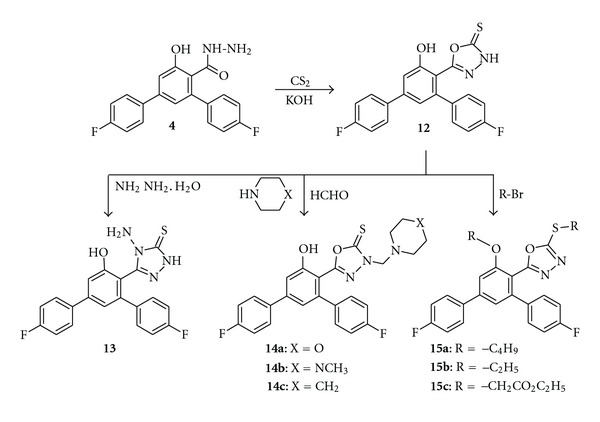


The treatment of hydrazides with carbon disulfide in the presence of alkali hydroxide was a general method leading to the formation of 1,3,4-oxadiazole-2-thione derivatives [27]. As the starting material, carbohydrazide **4** was used for the synthesis of 1,3,4-oxadiazole-2-thione derivative **12** (Scheme 4). The IR spectrum of compound **12** showed a sharp absorption band at 3224 cm^−1^ representing NH group. Its ^1^H NMR spectrum displayed a signal at *δ* 14.53 ppm due to the NH proton, while no signal representing a hydrazide structure appeared. The 4-amino-1,2,4-triazole-3-thione derivative **13** was synthesized starting from compound **12** by treating it with hydrazine hydrate. The IR spectrum of compound **13** showed two sharp absorption bands at 3373 cm^−1^ and 3259 cm^−1^ representing –NH and –NH_2_ groups. The singlet signal integrating two protons seen at *δ* 5.45 ppm in the ^1^H NMR spectrum of compound **13** was attributed to the –NH_2_ group. Furthermore, an additional singlet was observed due to the –NH group at *δ* 13.54 ppm integrating one proton.

Mannich reaction of 1,3,4-oxadiazole-2-thione derivative **12** with secondary amines and formaldehyde in absolute ethanol at room temperature yielded the desired compounds **14a–c**. When compared with the compound **12**, the IR spectra of Mannich bases **14a–c** showed the absence of NH group. Their ^1^H NMR spectra also supported the proposed structure as there was no signal corresponding to NH proton. Further, 1,3,4-oxadiazole-2-thione derivative **12** was alkylated using different alkyl halides. Compound **12** can exist in thione or thiol tautomeric forms. However, it has been established that under the action of alkyl halides *S*-substitution took place instead of *N*-substitution. The mass spectrum of **15c** showed a molecular ion peak at *m*/*z* 555.1 which reveals that the compound not only *S*-alkylated but also *O*-alkylated, because of the presence of phenolic group.

**Scheme 5:**
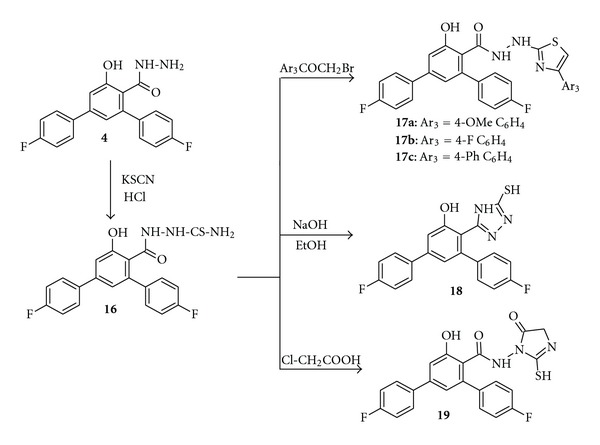


Furthermore, the intermediate thiosemicarbazide derivative  **16** representing a versatile building block for the synthesis of new heterocycles incorporating thiazole, imidazole, and triazole nucleus was synthesized by heating the carbohydrazide **4** with potassium thiocyanate in the presence of hydrochloric acid [28, 29] (Scheme 5). The ^1^H NMR spectrum of **16** exhibited singlet signals at *δ* 6.96 ppm and 8.19 ppm due to NH_2_ protons as one of the proton involved in intramolecular hydrogen bonding with carbonyl group; two protons were unequal. In addition to that, it showed two singlets at *δ* 9.57 and 10.45 ppm due to two NH protons. The IR spectrum of **16** exhibited two sharp absorption bands at 3464 cm^−1^ and 3329 cm^−1^ representing –NH and –NH_2_ groups. The thiazole derivatives **17a–c** was synthesized by the treatment of thiosemicarbazide derivative **16** with different phenacyl bromides in boiling ethanol. These reactions were assumed to proceed via *S*-alkylation followed by dehydration. The IR spectra of thiazoles **17a–c** showed absorption band at 3331 cm^−1^ representing –NH groups. Its ^1^H NMR spectra exhibited two singlets in the region *δ* 9.4–10.5 ppm for hydrazide (NH) protons, and hence confirming the formation of thiazole moiety.

Compound **16** was cyclized via oxidative cyclization in basic medium (10% NaOH), under reflux condition with subsequent acidification to give 1,2,4-triazole-3-thiol derivative **18**. Further, reflux of compound **16** with monochloroacetic acid and anhydrous sodium acetate in glacial acetic acid afforded a product identified as 2-imino-4-oxo-1,3-thiazolidine derivative **19** (Scheme 5). The structure of the synthesized products **18** and **19** was established on the basis of spectral data. For example, in compound **18**, the absence of absorption band of carbonyl group in IR spectrum and lack of ^1^H NMR resonances observed with NH and NH_2_ functions proved the intramolecular cyclization of compound **16** resulted in the formation of 1,2,4-triazole ring. Similarly, IR spectrum of thiazolidine derivative **19** showed an absorption band at 1724 cm^−1^ representing carbonyl group of imidazolidinone moiety. A singlet at *δ* 3.88 ppm present in the ^1^H NMR spectrum of **19** indicated the two protons of methylene group of imidazolidinone moiety, in addition to a singlet at *δ* 4.10 ppm due to SH proton and another singlet at *δ* 10.59 ppm due to that NH proton confirmed the presence of imidazolidinone moiety.

## 3. Biological Evaluation

### 3.1. Antibacterial Studies

The newly synthesized compounds were screened for their antibacterial activity against *Escherichia coli *(ATTC-25922), *Staphylococcus aureus *(ATTC-25923), *Pseudomonas aeruginosa *(ATCC-27853), and *Klebsiella pneumoniae *(recultured) bacterial strains by serial plate dilution method [30, 31]. Ciprofloxacin was used as standard antibiotics for antibacterial activity. 

Compound **12** was found to be the most active derivative against* Escherichia coli, Pseudomonas aeruginosa*, and *Klebsiella pneumoniae *at MIC value of 6.25 *μ*g/mL among the tested compounds and showed the same potency with ciprofloxacin. The compounds **3**, **4**, **5**, **6**, **16**, **17a**, **17b,** and **17c** were found to be active against all the tested microorganisms at MIC value of 12.5 *μ*g/mL. The high activity of **12** was due to the presence of 1,3,4-oxadiazole-2-thione moiety containing free NH group. Also the derivatives containing hydrazide moiety (**4**, **16**, **17a**, **17b**, **17c**), ester group (**3**), and pyrazole moiety (**5** & **6**) were helped to inhibit the growth of the microorganisms. 

### 3.2. Antifungal Studies

Newly prepared compounds were screened for their antifungal activity against *Aspergillus flavus *(NCIM No. 524), *Aspergillus fumigates *(NCIM No. 902), *Penicillium *(*S. aureus*) (recultured), and *Trichophyton mentagrophytes *(recultured) in DMSO by serial plate dilution method [32, 33]. Activity of each compound was compared with itraconazole as standard.

Compounds **6**, **8**, and **10b** were found to be the most active derivatives against all the tested microorganisms at an MIC value of 6.25 *μ*g/mL among the tested compounds, and showed the same potency with itraconozole. Although compounds **9a**, **9c**, **10a**, **11a**, **11b**, **11c**, **17a**, and **19** showed activity having MIC value of 12.5 *μ*g/mL against selected microorganisms, they exhibited lower potencies than the compared standard drug. Hence, it could be concluded that different functionality present in the molecules contributed to the antifungal activity in different manner comparable with that of standard drug tested. 

### 3.3. DPPH Radical Scavenging Assay

A rapid, simple, and inexpensive method to measure antioxidant capacity of substances involves the use of the free radical, 2,2-Diphenyl-1-picrylhydrazyl (DPPH). DPPH is widely used to test the ability of compounds to act as free radical scavengers or hydrogen donors. Antioxidants tested on DPPH were also found extremely effective in cell systems. This simple test further provides information on the ability of a compound to donate electrons during antioxidant action [34]. The radical scavenging mechanism is based on the transfer of acidic H-atom from the compound to DPPH radical to form DPPH-H. 

Among the tested compounds, many compounds showed significant radical scavenging capacity due to the presence of phenolic hydrogen in the parent molecule. Compounds **12** and **13** showed good radical scavenging capacity while compounds **9a**, **9b**, **14a**, **14b**, **15c**, and **16** exhibited moderate radical scavenging capacity with concentration of 1 mg/mL in comparison with the standard glutathione. The good radical scavenging capacity of compounds **12** and **13** was due to the presence of acidic protons in the oxadiazole moiety. The variation exhibited in DPPH scavenging capacity could be attributed to the effect of different moiety present in the compounds.

### 3.4. Reducing Power Assay

Reducing power is associated with antioxidant activity and may serve as a significant reflection of the antioxidant activity [35]. Compounds with reducing power indicate that they are electron donors and can reduce the oxidized intermediates of lipid peroxidation processes, so that they can act as primary and secondary antioxidants [36]. Substances, which have reduction potential, react with potassium ferricyanide (Fe^3+^) to form potassium ferrocyanide (Fe^2+^), which then reacts with ferric chloride to form ferrous complex that has an absorption maximum at 700 nm. Increased absorbance of the reaction mixture indicates the increased reducing power.

Reducing power assay is expressed in effective concentration (mg/mL) equivalent to 0.5 absorbance glutathione. Among the tested compounds, many compounds showed significant reducing power capacity. Compounds **4**, **12**, **13**, and **16** showed good reducing power capacity while compounds **5**, **8**, **10b**, **17a**, **17b**, **17c**, **18**, and **19** exhibited moderate reducing power capacity in comparison with the standard Glutathione. The good reducing power capacity of these compounds was due to the presence of free NH group in the molecules.

## 4. Conclusions

New functionalized terphenyl derivatives incorporating various heterocyclic rings are prepared by using 4,4′′-difluoro-5′-hydroxy-1,1′:3′,1′′-terphenyl-4′-carbohydrazide as a key intermediate derived from 4,4′-difluoro chalcone as a versatile synthone. All these derivatives are characterized by spectral data. All the synthesized products are screened for their *in vitro *antimicrobial and antioxidant properties. The majority of the tested compounds exhibited significant antimicrobial activity and some of them showed good DPPH scavenging activity. Hence this study has widened the scope of developing easy method to synthesize fluorinated functionalized terphenyl derivatives as promising antimicrobial and antioxidant agents.

## 5. Experimental

### 5.1. Chemistry

Melting points were taken in open capillary tubes and are uncorrected. The purity of the compounds was confirmed by thin layer chromatography using Merck silica gel 60 F_254_ coated aluminium plates. IR spectra were recorded on Shimadzu-FTIR Infrared spectrometer in KBr (*ν*_max_ in cm^−1^). ^1^HNMR (400 MHz) spectra were recorded on a Bruker AMX 400 spectrometer, with 5 mm PABBO BB-1H TUBES with TMS as internal standard. LCMS were obtained using Agilent 1200 series LC and Micromass zQ spectrometer. Elemental analysis was carried out by using VARIO EL-III (Elementar Analysensysteme GmBH). 

### 5.2. General Procedures for the Synthesis

**Synthesis of Ethyl 4,4′′-Difluoro-5′-hydroxy-1,1′:3′,1′′-terphenyl-4′-carboxylate (3)**

Ethyl 4,4′′-difluoro-5′-hydroxy-1,1′:3′,1′′-terphenyl-4′-carboxylate (**3**) was synthesized by the oxidative aromatization of ethyl 4,6-bis(4-fluorophenyl)-2-oxocyclohex-3-ene-1-carboxylate (**2**) using chloramine-T, which in turn was prepared by the condensation of ethyl acetoacetate to the 4,4′-difluoro chalcone (**1**) according to the methods described in our previous work [4].

**Synthesis of 4,4′′-Difluoro-5′-hydroxy-1,1′:3′,1′′-terphenyl-4′-carbohydrazide (4)**

A mixture of ester **3 **(17.7 g, 0.05 mol) and hydrazine hydrate (10 mL, 99%) in absolute ethanol (60 mL) was heated under reflux for 16 h and cooled at room temperature. The resultant solid was filtered and recrystallized from ethanol to give colorless crystals. Yield 86%; m.p 193–196°C. IR (KBr, *v* cm^−1^): 3290 (NH), 1672 (C=O), 1219 (C–F). ^1^H NMR (400 MHz, DMSO-d_6_, *δ* ppm): 4.18 (s, 2H, NH_2_), 6.99–7.69 (m, 10H, Ar–H), 9.19 (s, 1H, NH), 9.87 (s, 1H, OH). LCMS: *m*/*z* 341.0 (M^+^+1). C H N Analysis; calculated for C_19_H_14_F_2_N_2_O_2_: C, 67.05; H, 4.15; N, 8.23. Found: C, 67.02; H, 4.16; N, 8.20.

**Synthesis of (4,4′′-Difluoro-5′-hydroxy-1,1′:3′,1′′-terphenyl-4′-yl)(3,5-dimethyl-1H-pyrazol-1-yl)methanone (5)**

To a mixture of carbohydrazide **4** (3.4 g, 0.01 mol) and acetylacetone (1.0 g, 0.01 mol) in ethanol (20 mL) triethylamine (1 mL) was added, and the mixture was refluxed for 10 h. The precipitated solid was filtered off and recrystallized from ethanol. Yield 52%; m.p 166–168°C. IR (KBr, *v* cm^−1^): 3327 (OH), 2926 (C–H), 1604 (C=N), 1215 (C–F). ^1^H NMR (400 MHz, DMSO-d_6_, *δ* ppm): 2.29 (s, 3H, CH_3_), 2.70 (s, 3H, CH_3_), 6.28 (d, 1H, CH), 6.95–7.71 (m, 10H, Ar–H), 9.70 (d, 1H, OH). LCMS: *m*/*z* 407.0 (M^+^+1). C H N Analysis; calculated for C_24_H_20_F_2_N_2_O_2_: C, 71.28; H, 4.49; N, 6.93. Found: C, 71.24; H, 4.47; N, 6.91.

**Synthesis of 2-[(4,4′′-Difluoro-5′-hydroxy-1,1′:3′,1′′-terphenyl-4′-yl)carbonyl]-5-methyl-2,4-dihydro-3H-pyrazol-3-one (6)**

To a mixture of carbohydrazide **4** (3.4 g, 0.01 mol) and ethyl acetoacetate (1.3 g, 0.01 mol) in ethanol (20 mL) triethylamine (1 mL) was added, and the mixture was refluxed for 12 h. The precipitated solid was filtered off and recrystallized from ethanol. Yield 48%; m.p 96–98°C. IR (KBr, *v* cm^−1^): 3055 (OH), 2985 (C-H), 1654 (C=O), 1608 (C=N), 1211 (C–F). ^1^H NMR (400 MHz, DMSO-d_6_, *δ* ppm): 2.03 (s, 2H, CH_2_), 2.84 (s, 3H, CH_3_), 7.04–7.72 (m, 10H, Ar–H), 10.23 (s, 1H, OH). LCMS: *m*/*z* 407.1 (M^+^+1). C H N Analysis; calculated for C_23_H_16_F_2_N_2_O_3_: C, 67.98; H, 3.97; N, 6.89. Found: C, 67.94; H, 3.97; N, 6.84.

**Synthesis of 4,4′′-Difluoro-4′-(1,3,4-oxadiazol-2-yl)-1,1′:3′,1′′-terphenyl-5′-ol (7)**

A mixture of carbohydrazide **4** (3.4 g, 0.01 mol) and triethyl orthoformate (1 mL) in toluene (60 mL) was heated under reflux for 10 h. The excess solvent was evaporated and the reaction mass was cooled to room temperature. The resultant solid was filtered and recrystallized from ethanol. Yield 58%; m.p 193–196°C. IR (KBr, *v* cm^−1^): 3149 (OH), 1620 (C=N), 1232 (C–F). ^1^H NMR (400 MHz, DMSO-d_6_, *δ* ppm 7.12–7.79 (m, 10H, Ar–H), 9.23 (s, 1H, CH), 10.57 (s, 1H, OH). LCMS: *m*/*z* 351.0 (M^+^+1). C H N Analysis; calculated for C_20_H_12_F_2_N_2_O_2_: C, 68.57; H, 3.45; N, 8.00. Found: C, 68.54; H, 3.44; N, 7.98.

**Synthesis of 4′-(5-Amino-1,3,4-oxadiazol-2-yl)-4,4′′-difluoro-1,1′:3′,1′′-terphenyl-5′-ol (8)**

A mixture of carbohydrazide **4** (3.4 g, 0.01 mol) and cyanogen bromide (1.57 g, 0.015 mol) in absolute ethanol (20 mL) was refluxed for 5 h. The reaction mass was cooled to room temperature and neutralized with saturated sodium bicarbonate solution. The precipitated solid was filtered off and recrystallized from ethanol. Yield 61%; m.p 248–250°C. IR (KBr, *v* cm^−1^): 3385 (NH_2_), 3116 (OH), 1600 (C=N), 1217 (C–F). ^1^H NMR (400 MHz, DMSO-d_6_, *δ* ppm): 6.93 (s, 2H, NH_2_), 7.13–7.77 (m, 10H, Ar–H), 10.44 (s, 1H, OH). LCMS: *m*/*z* 366.0 (M^+^+1). C H N Analysis; calculated for C_20_H_13_F_2_N_3_O_2_: C, 65.75; H, 3.59; N, 11.50. Found: C, 65.73; H, 3.57; N, 11.47.

**Synthesis of 4,4′′-Difluoro-4′-(5-aryl-1,3,4-oxadiazol-2-yl)-1,1′:3′,1′′-terphenyl-5′-ol (9a–c)**

A mixture of carbohydrazide **4** (3.4 g, 0.01 mol) and aromatic carboxylic acid (0.015 mol) in phosphorus oxychloride (10 mL) was refluxed for 8 h. The excess phosphorus oxychloride was evaporated and the cooled reaction mass was then poured into ice cold water with stirring. The separated solid was filtered and washed with saturated sodium bicarbonate solution and then with water. The product was recrystallized from ethanol-dimethyl formamide mixture.

**4,4′′-Difluoro-4′-[5-(4-methoxyphenyl)-1,3,4-oxadiazol-2-yl]-1,1′:3′,1′′-terphenyl-5′-ol (9a)**

Yield 61%; m.p 198–201°C. IR (KBr, *v* cm^−1^): 3042 (OH), 2956 (CH), 1604 (C=N), 1220 (C–F). ^1^H NMR (400 MHz, DMSO-d_6_, *δ* ppm): 3.64 (s, 3H, CH_3_), 6.95–7.73 (m, 14H, Ar–H), 10.32 (s, 1H, OH). LCMS: *m*/*z* 457.1 (M^+^+1). C H N Analysis; calculated for C_27_H_18_F_2_N_2_O_3_: C, 71.05; H, 3.97; N, 6.14. Found: C, 71.03; H, 3.98; N, 6.12.

**4,4′′-Difluoro-4′-[5-(4-fluorophenyl)-1,3,4-oxadiazol-2-yl]-1,1′:3′,1′′-terphenyl-5′-ol (9b)**

Yield 66%; m.p 190–193°C. IR (KBr, *v* cm^−1^): 3032 (OH), 1604 (C=N), 1231 (C–F). ^1^H NMR (400 MHz, DMSO-d_6_, *δ* ppm): 6.98–7.70 (m, 14H, Ar–H), 9.85 (s, 1H, OH). LCMS: *m*/*z* 445.1 (M^+^+1). C H N Analysis; calculated for C_26_H_15_F_3_N_2_O_2_: C, 70.27; H, 3.40; N, 6.30. Found: C, 70.25; H, 3.42; N, 6.28.

**4,4′′-Difluoro-4′-[5-(biphenyl-4-yl)-1,3,4-oxadiazol-2-yl]-1,1′:3′,1′′-terphenyl-5′-ol (9c)**

Yield 68%; m.p 210–213°C. IR (KBr, *v* cm^−1^): 3039 (OH), 1606 (C=N), 1226 (C–F). ^1^H NMR (400 MHz, DMSO-d_6_, *δ* ppm): 7.13–8.11 (m, 19H, Ar–H), 10.13 (s, 1H, OH). LCMS: *m*/*z* 503.1 (M^+^+1). C H N Analysis; calculated for C_32_H_20_F_2_N_2_O_2_: C, 76.48; H, 4.01; N, 5.57. Found: C, 76.44; H, 4.02; N, 5.55.

**Synthesis of Schiff Base Derivatives (10a–c)**

A mixture of carbohydrazide **4** (3.4 g, 0.01 mol) and aromatic aldehyde (0.01 mol) in absolute ethanol (25 mL) was heated under reflux for 4 h in presence of concentrated HCl (0.5 mL). The reaction mixture was then poured into ice cold water and filtered. The pure product was recrystallized from ethanol-dimethyl formamide mixture.

**4,4′′-Difluoro-N′-[(Z)-(4-methoxyphenyl)methylidene]-5′-hydroxy-1,1′:3′,1′′-terphenyl-4′-carbohydrazide (10a)**

Yield 84%; m.p 177–180°C. IR (KBr, *v* cm^−1^): 3336 (NH), 3056 (OH), 1637 (C=O), 1606 (C=N), 1232 (C–F). ^1^H NMR (400 MHz, DMSO-d_6_, *δ* ppm): 3.21 (s, 3H, OCH_3_), 6.93–7.81 (m, 14H, Ar–H), 8.23 (s, 1H, CH), 10.32 (s, 1H, OH), 11.48 (s, 1H, NH). LCMS: *m*/*z* 459.1 (M^+^). C H N Analysis; calculated for C_27_H_20_F_2_N_2_O_3_: C, 70.73; H, 4.40; N, 6.11. Found: C, 70.71; H, 4.39; N, 6.08.

**4,4′′-Difluoro-N′-[(Z)-(4-fluorophenyl)methylidene]-5′-hydroxy-1,1′:3′,1′′-terphenyl-4′-carbohydrazide (10b)**

Yield 87%; m.p 108–110°C. IR (KBr, *v* cm^−1^): 3354 (NH), 3046 (OH), 1640 (C=O), 1600 (C=N), 1223 (C–F). ^1^H NMR (400 MHz, DMSO-d_6_, *δ* ppm): 6.91–7.78 (m, 14H, Ar–H), 7.93 (s, 1H, CH), 9.85 (s, 1H, OH), 10.91 (s, 1H, NH). LCMS: *m*/*z* 447.1 (M^+^). C H N Analysis; calculated for C_26_H_17_F_3_N_2_O_2_: C, 69.95; H, 3.84; N, 6.28. Found: C, 69.94; H, 3.86; N, 6.24.

**4,4′′-Difluoro-N′-[(Z)-(biphenyl-4-yl)methylidene]-5′-hydroxy-1,1′:3′,1′′-terphenyl-4′-carbohydrazide (10c)**

Yield 82%; m.p 226–228°C. IR (KBr, *v* cm^−1^): 3329 (NH), 3059 (OH), 1647 (C=O), 1604 (C=N), 1217 (C–F). ^1^H NMR (400 MHz, DMSO-d_6_, *δ* ppm): 7.07–7.97 (m, 19H, Ar–H), 8.15 (s, 1H, CH), 10.15 (s, 1H, OH), 11.71 (s, 1H, NH). LCMS: *m*/*z* 505.1 (M^+^+1). C H N Analysis; calculated for C_32_H_22_F_2_N_2_O_2_: C, 76.18; H, 4.40; N, 5.55. Found: C, 76.14; H, 4.38; N, 5.52.

**Acetylation of Schiff Base Derivatives (11a–c)**

A mixture of Schiff base (0.01 mol) and acetic anhydride (10 mL) was dissolved in ethanol (25 mL) and the reaction mixture was refluxed for 6 h. The reaction mixture was then concentrated and allowed to cool. The solid product obtained was filtered, washed with water, and recrystallized using ethanol-dimethyl formamide mixture.

**1-[5-(4,4′′-Difluoro-5′-hydroxy-1,1′:3′,1′′-terphenyl-4′-yl)-2-(4-methoxyphenyl)-1,3,4-oxadiazol-3(2H)-yl]ethanone (11a)**

Yield 42%; m.p 124–126°C. IR (KBr, *v* cm^−1^): 3059 (OH), 2933, 2837 (CH), 1770 (C=O), 1608 (C=N), 1224 (C–F). ^1^H NMR (400 MHz, DMSO-d_6_, *δ* ppm): 1.84 (s, 3H, COCH_3_), 3.45 (s, 3H, OCH_3_), 6.41 (s, 1H, CH), 6.74–7.85 (m, 14H, Ar–H), 9.98 (s, 1H, OH). LCMS: *m*/*z* 501.1 (M^+^+1). C H N Analysis; calculated for C_29_H_22_F_2_N_2_O_4_: C, 69.59; H, 4.43; N, 5.60. Found: C, 69.57; H, 4.44; N, 5.58.

**1-[5-(4,4′′-Difluoro-5′-hydroxy-1,1′:3′,1′′-terphenyl-4′-yl)-2-(4-fluorophenyl)-1,3,4-oxadiazol-3(2H)-yl]ethanone (11b)**

Yield 45%; m.p 142–145°C. IR (KBr, *v* cm^−1^): 3043 (OH), 2889 (CH), 1762 (C=O), 1604 (C=N), 1232 (C–F). ^1^H NMR (400 MHz, DMSO-d_6_, *δ* ppm): 1.72 (s, 3H, COCH_3_), 6.52 (s, 1H, CH), 6.82–7.91 (m, 14H, Ar–H), 9.85 (s, 1H, OH). LCMS: *m*/*z* 489.1 (M^+^). C H N Analysis; calculated for C_28_H_19_F_3_N_2_O_3_: C, 68.85; H, 3.92; N, 5.74. Found: C, 68.83; H, 3.90; N, 5.73.

**1-[2-(Biphenyl-4-yl)-5-(4,4′′-difluoro-5′-hydroxy-1,1′:3′,1′′-terphenyl-4′-yl)-1,3,4-oxadiazol-3(2H)-yl]ethanone (11c)**

Yield 50%; m.p 133–126°C. IR (KBr, *v* cm^−1^): 3032 (OH), 2915 (CH), 1742 (C=O), 1611 (C=N), 1215 (C–F). ^1^H NMR (400 MHz, DMSO-d_6_, *δ* ppm): 1.94 (s, 3H, COCH_3_), 6.48 (s, 1H, CH), 7.03–7.99 (m, 19H, Ar–H), 10.15 (s, 1H, OH). LCMS: *m*/*z* 547.1 (M^+^). C H N Analysis; calculated for C_34_H_24_F_2_N_2_O_3_: C, 74.71; H, 4.43; N, 5.13. Found: C, 74.72; H, 4.42; N, 5.10.

**Synthesis of 5-(4,4′′-Difluoro-5′-hydroxy-1,1′:3′,1′′-terphenyl-4′-yl)-1,3,4-oxadiazole-2(3H)-thione (12)**

To a solution of carbohydrazide **4** (3.4 g, 0.01 mol) in ethanol (10 mL) a solution of carbon disulphide (1.14 g, 0.015 mol) in water (3 mL) and potassium hydroxide (1 g) was added, and the mixture was refluxed until the evolution of H_2_S ceased (7-8 h). The reaction mixture was allowed to cool and then acidified with dilute hydrochloric acid. The solid obtained was collected by filtration, washed with least amount of water, and recrystallized from ethanol-water (1 : 1). Yield 62%; m.p 121–124°C. IR (KBr, *v* cm^−1^): 3224 (NH), 3124 (OH), 1604(C=N), 1228 (C–F). ^1^H NMR (400 MHz, DMSO-d_6_, *δ* ppm): 7.17–7.78 (m, 10H, Ar–H), 10.75 (s, 1H, OH), 14.53 (s, 1H, NH). LCMS: *m*/*z* 383.0 (M^+^+1). C H N Analysis; calculated for C_20_H_12_F_2_N_2_O_2_S: C, 62.82; H, 3.16; N, 7.33. Found: C, 62.80; H, 3.17; N, 7.30.

**Synthesis of 4-Amino-5-(4,4′′-difluoro-5′-hydroxy-1,1′:3′,1′′-terphenyl-4′-yl)-2,4-dihydro-3H-1,2,4-triazole-3-thione (13)**

A mixture of the oxadiazole **12** (3.82 g, 0.01 mol) and hydrazine hydrate (5 mL, 95%) in water (20 mL) was refluxed with stirring for 4 h, diluted with cold water (200 mL), acidified by the drop wise addition of concentrated hydrochloric acid, and filtered off. The solid formed was washed with the least amount of cold water and recrystallized from ethanol-dimethyl formamide mixture. Yield 58%; m.p 240–242°C. IR (KBr, *v* cm^−1^): 3373 (NH_2_), 3259 (NH), 3163 (OH), 1616 (C=N), 1219 (C–F). ^1^H NMR (400 MHz, DMSO-d_6_, *δ* ppm): 5.45 (s, 2H, NH_2_), 7.11–7.76 (m, 10H, Ar–H), 10.43 (s, 1H, OH), 13.54 (s, 1H, NH). LCMS: *m*/*z* 397.0 (M^+^+1). C H N Analysis; calculated for C_20_H_14_F_2_N_4_OS: C, 60.60; H, 3.56; N, 14.13. Found: C, 60.56; H, 3.57; N, 14.10.

**Synthesis of Mannich Bases (14a–c)**

To a solution of oxadiazole **12** (3.82 g, 0.01 mole) in ethanol (5 mL), formaldehyde (0.5 mL, 37%) and secondary amine (0.01 mol) were added. The reaction mixture was stirred overnight. After cooling, the precipitate was filtered and recrystallized from ethanol.

**5-(4,4′′-Difluoro-5′-hydroxy-1,1′:3′,1′′-terphenyl-4′-yl)-3-(morpholin-4-ylmethyl)-1,3,4-oxadiazole-2(3H)-thione (14a)**

Yield 72%; m.p 200–202°C. IR (KBr, *v* cm^−1^): 3157 (OH), 2937, 2858 (CH), 1620 (C=N), 1236 (C–F). ^1^H NMR (400 MHz, DMSO-d_6_, *δ* ppm): 2.35 (t, 4H, N-CH_2_ of morpholine), 3.45 (t, 4H, O–CH_2_ of morpholine), 4.90 (s, 2H, N–CH_2_–N), 7.19–7.79 (m, 10H, Ar–H), 10.83 (s, 1H, OH). C H N Analysis; calculated for C_25_H_21_F_2_N_3_O_3_S: C, 62.36; H, 4.40; N, 8.73. Found: C, 62.34; H, 4.41; N, 8.71.

**5-(4,4′′-Difluoro-5′-hydroxy-1,1′:3′,1′′-terphenyl-4′-yl)-3-[(4-methylpiperazin-1-yl)methyl]-1,3,4-oxadiazole-2(3H)-thione (14b)**

Yield 68%; m.p 191–194°C. IR (KBr, *v* cm^−1^): 3163 (OH), 2862 (CH), 1616 (C=N), 1219 (C–F). ^1^H NMR (400 MHz, DMSO-d_6_, *δ* ppm): 2.29 (s, 3H, N–CH_3_), 2.45 (t, 2H, CH_2_), 2.95 (t, 4H, CH_2_), 5.07 (s, 2H, N–CH_2_–N), 7.17–7.89 (m, 10H, Ar–H), 10.72 (s, 1H, OH). C H N Analysis; calculated for C_26_H_24_F_2_N_4_O_2_S: C, 63.14; H, 4.89; N, 11.33. Found: C, 63.12; H, 4.87; N, 11.30.

**5-(4,4′′-Difluoro-5′-hydroxy-1,1′:3′,1′′-terphenyl-4′-yl)-3-(piperidin-1-ylmethyl)-1,3,4-oxadiazole-2(3H)-thione (14c)**

Yield 61%; m.p 98–100°C. IR (KBr, *v* cm^−1^): 3173 (OH), 2912 (CH), 1608 (C=N), 1222 (C–F). ^1^H NMR (400 MHz, DMSO-d_6_, *δ* ppm): 1.55–1.61 (m, 6H, CH_2_), 2.82 (t, 4H, N–CH_2_), 5.23 (s, 2H, N–CH_2_–N), 7.11–7.76 (m, 10H, Ar–H), 10.63 (s, 1H, OH). C H N Analysis; calculated for C_26_H_23_F_2_N_3_O_2_S: C, 65.12; H, 4.83; N, 8.76. Found: C, 65.10; H, 4.84; N, 8.74.

**Alkylation of 1,3,4-Oxadiazole-2-thione Derivative (15a–c)**

Oxadiazole **12** (3.82 g, 0.01 mol) was dissolved in ethanol (20 mL) and 10% aqueous sodium hydroxide solution (3.5 mL) was added. Alkyl halide (0.02 mol) was added and heated to reflux for 4 h. The reaction mixture was then poured into ice cold water, filtered, and crystallized from ethanol.

**5-(Butylsulfanyl)-2-(5′-butoxy-4,4′′-difluoro-1,1′:3′,1′′-terphenyl-4′-yl)-1,3,4-oxadiazole (15a)**

Yield 63%; m.p 270–272°C. IR (KBr, *v* cm^−1^): 2964 (CH), 1560(C=N), 1170 (C–F). LCMS: *m*/*z* 494.3 (M^+^). C H N Analysis; calculated for C_28_H_28_F_2_N_2_O_2_S: C, 67.99; H, 5.71; N, 5.66. Found: C, 67.96; H, 5.69; N, 5.63.

**2-(4,4′′-Difluoro-5′-ethoxy-1,1′:3′,1′′-terphenyl-4′-yl)- 5-(Ethylsulfanyl)-1,3,4-oxadiazole (15b)**

Yield 58%; m.p 222–224°C. IR (KBr, *v* cm^−1^): 2972 (CH), 1572(C=N), 1220 (C–F). LCMS: *m*/*z* 438.4 (M^+^). C H N Analysis; calculated for C_24_H_20_F_2_N_2_O_2_S: C, 65.74; H, 4.60; N, 6.39. Found: C, 65.71; H, 4.57; N, 6.35.

**Ethyl ({5-[4,4′′-difluoro-5′-(2-ethoxy-2-oxoethoxy)-1,1′:3′,1′′-terphenyl-4′-yl]-1,3,4-oxadiazol-2-yl}sulfanyl)acetate (15c)**

Yield 72%; m.p 103–105°C. IR (KBr, *v* cm^−1^): 2954 (CH), 1724 (C=O ester), 1220 (C–F). ^1^H NMR (400 MHz, DMSO-d_6_, *δ* ppm): 1.14 (m, 6H, 2 × CH_3_), 3.34 (s, 2H, SCH_2_), 4.09 (m, 4H, 2 × CH_2_), 5.05 (s, 2H, OCH_2_), 7.16–7.91 (m, 10H, Ar–H). LCMS: *m*/*z* 555.1 (M^+^+1). C H N Analysis; calculated for C_28_H_24_F_2_N_2_O_6_S: C, 60.64; H, 4.36; N, 5.05. Found: C, 60.61; H, 4.333; N, 5.01.

**Synthesis of 2-[(4,4′′-Difluoro-5′-hydroxy-1,1′:3′,1′′-terphenyl-4′-yl)carbonyl]hydrazine carbothioamide (16)**

A mixture of carbohydrazide **4** (3.4 g, 0.01 mol), potassium thiocyanate (0.015 mol), and hydrochloric acid (5 mL) in water (25 mL) was refluxed for 4 h with stirring. The resulting solid that separated was washed with hot water and recrystallized from 75% ethanol. Yield 83%; m.p 200–202°C. IR (KBr, *v* cm^−1^): 3464, 3329 (NH), 3236 (OH), 1672 (C=O), 1608 (C=N), 1226 (C–F). ^1^H NMR (400 MHz, DMSO-d_6_, *δ* ppm): 6.96, 8.19 (two singlets, 2H, NH_2_), 7.09–7.71 (m, 10H, Ar–H), 9.57 (s, 1H, NH), 10.45 (s, 1H, NH), 10.75 (s, 1H, OH). LCMS: *m*/*z* 400.0 (M^+^+1). C H N Analysis; calculated for C_20_H_15_F_2_N_3_O_2_S: C, 60.14; H, 3.79; N, 10.52. Found: C, 60.11; H, 3.79; N, 10.50.

**Synthesis of 1,3-Thiazol Derivatives (17a–c)**

An equimolar mixture of thiosemicarbazide derivative **16** (0.399 g, 0.001 mol) and phenacyl bromide derivative (0.001 mol) in 20 mL of absolute ethanol was heated under reflux for 4 h. The product that separated after cooling was collected and recrystallized from ethanol.

**4,4′′-Difluoro-5′-hydroxy-N′-[4-(4-methoxyphenyl)-1,3-thiazol-2-yl]-1,1′:3′,1′′-terphenyl-4′-carbohydrazide (17a)**

Yield 64%; m.p 213–215°C. IR (KBr, *v* cm^−1^): 3331(NH), 3196 (OH), 1654 (C=O), 1606 (C=N), 1228 (C–F). ^1^H NMR (400 MHz, DMSO-d_6_, *δ* ppm): 3.75 (s, 3H, OCH_3_), 6.91–7.73 (m, 14H, Ar–H), 9.47 (s, 1H, NH), 10.23 (s, 1H, NH), 10.37 (s, 1H, OH). LCMS: *m*/*z* 530.1 (M^+^+1). C H N Analysis; calculated for C_29_H_21_F_2_N_3_O_3_S: C, 65.77; H, 4.00; N, 7.93. Found: C, 65.74; H, 3.96; N, 7.90.

**4,4′′-Difluoro-5′-hydroxy-N′-[4-(4-fluorophenyl)-1,3-thiazol-2-yl]-1,1′:3′,1′′-terphenyl-4′-carbohydrazide (17b)**

Yield 67%; m.p 200–202°C. IR (KBr, *v* cm^−1^): 3329 (NH), 3236 (OH), 1662 (C=O), 1608 (C=N), 1226 (C–F). ^1^H NMR (400 MHz, DMSO-d_6_, *δ* ppm): 6.81–7.67 (m, 14H, Ar–H), 9.39 (s, 1H, NH), 10.17 (s, 1H, NH), 10.41 (s, 1H, OH). LCMS: *m*/*z* 518.2 (M^+^+1). C H N Analysis; calculated for C_28_H_18_F_3_N_3_O_2_S: C, 64.98; H, 3.51; N, 8.12. Found: C, 64.96; H, 3.50; N, 8.08.

**4,4′′-Difluoro-5′-hydroxy-N′-[4-(biphenyl-4-yl)-1,3-thiazol-2-yl]-1,1′:3′,1′′-terphenyl-4′-carbohydrazide (17c)**

Yield 60%; m.p 169–172°C. IR (KBr, *v* cm^−1^): 3324 (NH), 3214 (OH), 1652 (C=O), 1601 (C=N), 1221 (C–F). ^1^H NMR (400 MHz, DMSO-d_6_, *δ* ppm): 6.69–8.14 (m, 19H, Ar–H), 9.41 (s, 1H, NH), 10.29 (s, 1H, NH), 10.74 (s, 1H, OH). LCMS: *m*/*z* 576.3 (M^+^+1). C H N Analysis; calculated for C_34_H_23_F_2_N_3_O_2_S: C, 70.94; H, 4.03; N, 7.30. Found: C, 70.91; H, 4.01; N, 7.27.

**Synthesis of 4,4′′-Difluoro-4′-(5-sulfanyl-4H-1,2,4-triazol-3-yl)-1,1′:3′,1′′-terphenyl-5′-ol (18)**

A suspension of the thiosemicarbazide derivative **16** (0.399 g, 0.001 mol) in sodium hydroxide solution (10 mL, 10%) was heated under reflux for 10 h. The reaction mixture was allowed to cool and then adjusted to pH 6 with 10% hydrochloric acid. The precipitate that formed was filtered off, washed with water, dried, and recrystallized from ethanol. Yield 60%; m.p 189–192°C. IR (KBr, *v* cm^−1^): 3433, 3329 (NH), 3236 (OH), 2372 (SH), 1608 (C=N), 1228 (C–F). ^1^H NMR (400 MHz, DMSO-d_6_, *δ* ppm): 4.07 (s, 1H, SH), 7.05–7.72 (m, 10H, Ar–H), 10.23 (s, 1H, OH), 13.54 (s, 1H, NH). LCMS: *m*/*z* 382.0 (M^+^+1). C H N Analysis; calculated for C_20_H_13_F_2_N_3_OS: C, 62.98; H, 3.44; N, 11.02. Found: C, 62.94; H, 3.42; N, 10.99.

**Synthesis of 4,4′′-Difluoro-5′-hydroxy-N-(5-oxo-2-sulfanyl-4,5-dihydro-1H-imidazol-1-yl)-1,1′:3′,1′′-terphenyl-4′-carboxamide (19)**

A mixture of thiosemicarbazide derivative **16** (0.399 g, 0.001 mol), monochloroacetic acid (0.001 mol), and anhydrous sodium acetate (0.002 mol) in glacial acetic acid (30 mL) was refluxed for 8 h. After cooling, the reaction mixture was poured on crushed ice. The resulting solid that separated was washed with hot water and recrystallized from ethanol. Yield 65%; m.p 262–264°C. IR (KBr, *v* cm^−1^): 3327 (NH), 3132 (OH), 1724, 1635 (C=O), 1598 (C=N), 1222 (C–F). ^1^H NMR (400 MHz, DMSO-d_6_, *δ* ppm): 3.88 (s, 2H, CH_2_), 4.10 (s, 1H, SH), 6.94–7.78 (m, 10H, Ar–H), 10.59 (s, 1H, NH), 10.60 (s, 1H, OH). LCMS: *m*/*z* 440.0 (M^+^+1). C H N Analysis; calculated for C_22_H_15_F_2_N_3_O_3_S: C, 60.13; H, 3.44; N, 9.56. Found: C, 60.10; H, 3.42; N, 9.52.

### 5.3. Biological Evaluation 

#### 5.3.1. Antibacterial Studies

The newly synthesized compounds were screened for their antibacterial activity against *Escherichia coli *(ATTC-25922), *Staphylococcus aureus *(ATTC-25923), *Pseudomonas aeruginosa *(ATCC-27853), and *Klebsiella pneumoniae *(recultured) bacterial strains by serial plate dilution method. Serial dilutions of the drug in Mueller-Hinton broth were taken in tubes and their pH was adjusted to 5.0 using phosphate buffer. A standardized suspension of the test bacterium was inoculated and incubated for 16–18 h at 37°C. The minimum inhibitory concentration (MIC) was noted by seeing the lowest concentration of the drug at which there was no visible growth. A number of antimicrobial discs were placed on the agar for the sole purpose of producing zones of inhibition in the bacterial lawn. About 20 mL of agar media was poured into each petri dish. Excess of suspension was decanted and plates were dried by placing in an incubator at 37°C for an hour. Using a punch, wells were made on these seeded agar plates and minimum inhibitory concentrations of the test compounds in dimethylsulfoxide (DMSO) were added into each labeled well. A control was also prepared for the plates in the same way using solvent DMSO. The petri dishes were prepared in triplicate and maintained at 37°C for 3-4 days. Antibacterial activity was determined by measuring the diameter of inhibition zone. Activity of each compound was compared with ciprofloxacin as standard. Zone of inhibition was determined for newly synthesized compounds and the results are summarized in Table 1.

#### 5.3.2. Antifungal Studies

Newly prepared compounds were screened for their antifungal activity against *Aspergillus flavus *(NCIM No. 524), *Aspergillus fumigates *(NCIM No. 902), *Penicillium *(*S. aureus*) (recultured), and *Trichophyton mentagrophytes *(recultured) in DMSO by serial plate dilution method. Sabourands agar media were prepared by dissolving peptone (1 g), D-glucose (4 g) and agar (2 g) in distilled water (100 mL) and adjusting the pH to 5.7. Normal saline was used to make a suspension of spore of fungal strains for lawning. A loopful of particular fungal strain was transferred to 3 mL saline to get a suspension of corresponding species. Twenty milliliters of agar media was poured into each Petri dish. Excess of suspension were decanted and plates were dried by placing in incubator at 37°C for 1 h. Using a punch, wells were made on these seeded agar plates minimum inhibitory concentrations of the test compounds in DMSO were added into each labeled well. A control was also prepared for the plates in the same way using solvent DMSO. The Petri dishes were prepared in triplicate and maintained at 37°C for 3-4 days. Antifungal activity was determined by measuring the diameter of inhibition zone. Activity of each compound was compared with itraconozole as standard. Zones of inhibition were determined and the results are summarized in Table 2.

#### 5.3.3. DPPH Radical Scavenging Assay

The DPPH assay was based on the reported method [37]. Briefly, 1 mM solution of DPPH in ethanol was prepared, and this solution (1 mL) was added to sample solutions 1 mg/mL of DMSO. The mixture was shaken vigorously and allowed to stand at room temperature for 20 min. Then the absorbance was measured at 517 nm in a spectrophotometer. Lower absorbance of the reaction mixture indicated higher free radical scavenging activity. The capability to scavenge the DPPH radical was calculated using the following equation:

(1) $$\begin{array}{c} \text{DPPH}\;\;\text{scavenging}\;\;\text{effect}\;\;\left(\%\right)=\left(A_{0}-\frac{A_{1}}{A_{0}}\right)\times 100, \end{array}$$

where *A*_0_ is the absorbance of the control reaction and *A*_1_ is the absorbance in the presence of the samples or standards. Each sample was assayed at 1 mg/mL and all experiments were carried out in triplicate and the % RSC is shown in Table 3.

#### 5.3.4. Reducing Power Assay

The reducing power of the synthesized compounds was determined according to the method of Oyaizu [38]. Different concentrations of the samples (100–1000 *μ*g/mL) in DMSO (1 mL) were mixed with phosphate buffer (2.5 mL, 0.2 M, pH = 6.6) and potassium ferricyanide (2.5 mL, 1% solution). The mixture was incubated at 50°C for 20 min. after which 10% trichloroacetic acid (2.5 mL) was added to the mixture, which was then centrifuged for 10 min. The upper layer of solution (2.5 mL) was mixed with distilled water (2.5 mL) and FeCl_3_ (0.5 mL, 0.1%) and then the absorbance at 700 nm was measured using a spectrophotometer. Higher absorbance of the reaction mixture indicated greater reducing power. All experiments were carried out in triplicate and the reducing power assay was represented by effective concentration (mg/mL) equivalent to 0.5 absorbance glutathione (Table 3).

## Figures and Tables

**Table 1:** Antibacterial activity of the synthesized compounds: MIC in *μ*g/mL (Zone of inhibition in mm).

Compound	*St* *ap* *hy* *lo* *co* *cc* *us* *aureus*	*Es* *ch* *er* *i* *ch* *i* *a* *coli*	*Ps* *eu* *do* *mo* *na* *s* *Aeruginosa*	*Kl* *eb* *si* *el* *la* *pneumonia*
	(ATTC-25923)	(ATTC-25922)	(ATTC-27853)	(recultured)
**3**	12.5 (6)	12.5 (6)	12.5 (6)	12.5 (6)
**4**	12.5 (6)	12.5 (8)	12.5 (6)	12.5 (6)
**5**	12.5 (6)	12.5 (6)	12.5 (6)	12.5 (8)
**6**	12.5 (6)	12.5 (6)	12.5 (6)	12.5 (6)
**7**	R	100 (2)	100 (4)	100 (5)
**8**	R	R	R	R
**9a**	R	R	R	R
**9b**	R	R	R	R
**9c**	R	R	R	R
**10a**	R	R	R	R
**10b**	R	R	R	R
**10c**	R	R	R	R
**11a**	R	R	R	R
**11b**	R	R	R	R
**11c**	R	R	R	R
**12**	100 (4)	6.25 (6)	6.25 (6)	6.25 (6)
**13**	R	R	R	R
**14a**	R	R	R	R
**14b**	R	R	R	R
**14c**	R	R	R	R
**15a**	R	R	R	R
**15b**	R	R	R	R
**15c**	R	R	R	R
**16**	12.5 (6)	12.5 (6)	12.5 (6)	12.5 (6)
**17a**	12.5 (6)	12.5 (6)	12.5 (6)	12.5 (6)
**17b**	R	12.5 (6)	12.5 (6)	12.5 (6)
**17c**	12.5 (6)	12.5 (6)	12.5 (6)	12.5 (6)
**18**	R	R	R	R
**19**	R	R	R	R
**Ciprofloxacin**	6.25 (20)	6.25 (17)	6.25 (19)	6.25 (18)

Note: R indicates bacterial strains are resistant to the compounds >100 *μ*g/mL.

**Table 2:** Antifungal activity of the synthesized compounds: MIC in *μ*g/mL (Zone of inhibition in mm).

Compound	*Penicillium marneffei *	*Trichophyton mentagrophytes *	*Aspergillus flavus *	*Aspergillus fumigates *
	(recultured)	(recultured)	(NCIM No. 524)	(NCIM No. 902)
**3**	R	R	R	R
**4**	R	R	R	R
**5**	R	R	R	R
**6**	6.25 (8)	6.25 (8)	6.25 (8)	6.25 (8)
**7**	R	R	R	R
**8**	6.25 (8)	6.25 (8)	6.25 (8)	6.25 (8)
**9a**	100 (5)	12.5 (6)	12.5 (6)	R
**9b**	R	R	R	R
**9c**	100 (2)	12.5 (6)	12.5 (6)	R
**10a**	12.5 (6)	R	R	R
**10b**	6.25 (8)	6.25 (8)	6.25 (8)	6.25 (8)
**10c**	R	R	R	R
**11a**	100 (4)	12.5 (6)	12.5 (6)	R
**11b**	12.5 (6)	12.5 (6)	12.5 (6)	R
**11c**	100 (3)	12.5 (6)	12.5 (6)	R
**12**	R	R	R	R
**13**	R	R	R	R
**14a**	R	R	R	R
**14b**	R	R	R	R
**14c**	R	R	R	R
**15a**	R	R	R	R
**15b**	R	R	R	R
**15c**	R	R	R	R
**16**	R	R	R	R
**17a**	12.5 (6)	12.5 (6)	12.5 (6)	12.5 (6)
**17b**	R	R	R	R
**17c**	12.5 (6)	R	R	R
**18**	R	R	R	R
**19**	R	12.5 (8)	12.5 (6)	12.5 (8)
**Itraconozole**	6.25 (17)	6.25 (19)	6.25 (18)	6.25 (18)

Note: R indicates fungal strains are resistant to the compounds >100 *μ*g/mL.

**Table 3:** Antioxidant activity of synthesized compounds.

Compounds	% DPPH scavenging	Reducing power assay
**3**	8.46 ± 4.49	18.18 ± 0.46
**4**	48.40 ± 1.12	0.78 ± 0.01
**5**	35.44 ± 10.47	6.39 ± 1.52
**6**	28.30 ± 1.12	10.32 ± 0.45
**7**	10.05 ± 2.73	11.90 ± 0.01
**8**	29.09 ± 1.42	6.15 ± 1.25
**9a**	69.54 ± 4.35	14.43 ± 3.96
**9b**	54.94 ± 3.14	10.45 ± 0.92
**9c**	42.32 ± 5.35	12.11 ± 2.14
**10a**	7.66 ± 1.85	10.22 ± 0.59
**10b**	15.34 ± 2.23	6.99 ± 1.16
**10c**	19.04 ± 5.97	8.23 ± 2.22
**11a**	36.81 ± 0.64	63.49 ± 11.22
**11b**	32.80 ± 0.01	10.82 ± 3.31
**11c**	47.27 ± 3.86	21.47 ± 16.77
**12**	72.22 ± 2.62	1.49 ± 0.11
**13**	73.63 ± 9.00	1.65 ± 0.19
**14a**	61.36 ± 9.64	7.82 ± 1.46
**14b**	57.26 ± 6.42	20.51 ± 8.21
**14c**	49.54 ± 3.07	8.77 ± 1.18
**15a**	1.01 ± 0.01	26.06 ± 4.72
**15b**	19.99 ± 3.29	8.25 ± 0.96
**15c**	68.63 ± 9.64	7.67 ± 2.23
**16**	51.58 ± 4.11	1.12 ± 0.07
**17a**	16.43 ± 2.54	6.18 ± 1.87
**17b**	49.09 ± 4.99	4.29 ± 0.54
**17c**	27.50 ± 4.49	6.07 ± 1.66
**18**	22.22 ± 7.48	2.16 ± 0.25
**19**	15.40 ± 2.91	5.92 ± 1.44
**Glutathione **	92.09 ± 1.09	0.595 ± 0.01
